# Supplementary material for: Branched chain amino acid metabolism and microbiome in adolescents with obesity during weight loss therapy
Source: J Clin Invest. 2026 Jul 15;136(14):e196742. doi: 10.1172/JCI196742 (PMC13367979; doi:10.1172/JCI196742)
Supplement: Supplemental data [file jci-136-196742-s112.pdf]

Supplemental Methods

Serum metabolomics: Serum metabolomic samples from the Hearts and Parks study (1) were randomized and analyzed with the POMMS cohort samples to reduce drift from batch effects. Pooled samples were run with each batch, and potential batch variation slope was measured and determined to be not significantly different from 0 (Supplemental Figure 8). All serum metabolomics assays were performed at the Duke Molecular Physiology Institute. Serum samples were randomized and analyzed for the conventional metabolites nonesterified fatty acids (NEFA), glucose, insulin, glycerol, blood urea nitrogen (BUN), 3-hydroxybutyrate (3-HB), total ketones, triglycerides, and uric acid. The samples were run in seven daily batches of approximately 76 sera per batch. Samples were run on a Beckman autoanalyzer from individual tubes, with pooled serum controls inserted at the beginning, middle, and end of the sequence. Reagents for measuring for total ketones, 3-HB, and NEFA were from Fujifilm (Osaka, Japan). Samples were additionally assayed for insulin using kits from Meso Scale Discovery (Rockville, MD). All samples were refrozen afterwards for later insulin immunoassays on 96-well plates. Corresponding to each batch of metabolites were two insulin plates (e.g. 1-2 for Batch 1, 3-4 for Batch 2, and so on) on which the 76 samples were assayed, 38 per plate. Control pooled serum samples were run twice on each insulin plate, placed at the beginning and end. Insulin was measured by immunoassay using reagents and electrochemiluminescent imager from Meso Scale Discovery (Rockville, MD). Glucose was measured on a Beckman DxC 600 clinical analyzer (Brea, CA). HOMA-IR was calculated as (fasting insulin in units/liter x fasting glucose in mg/deciliter)/405 (2).

Serum samples were analyzed for amino acids and acylcarnitines in a targeted assay using labeled standards as described (3). BCKA were analyzed by LC-MS/MS as described (4). Briefly, 20 µl of plasma containing isotopically labeled internal standards KIC-d3, KIV-5C13 (Cambridge Isotope Laboratories), and KMV-d8 (Toronto Research Chemicals) was precipitated with 150 µl of 3M PCA. 200 µl of 25 M *o*-phenylenediamine (OPD) in 3M HCl was added to the supernatants, and the samples were incubated at 80°C for 20 minutes. Keto acids were extracted with ethyl acetate as previously described (5). The extracts were dried under nitrogen, reconstituted in 200 mM ammonium acetate, and analyzed on a Waters Xevo TQ-S triple quadrupole mass spectrometer coupled to a Waters Acquity UPLC system. The analytical column (Waters Acquity UPLC BEH C18 Column, 1.7 µm, 2.1 × 50 mm) was used at 30°C. 10 µl of the sample was injected onto the column and eluted at a flow rate of 0.4 ml/min. The gradient consisted of 45% eluent A (5 mM ammonium acetate in water) and 55% eluent B (methanol) for 2 min., followed by a linear gradient to 95% B from 2 to 2.5 min., held at 95% B for 0.7 min., returned to 45% A, and then the column was re-equilibrated at initial conditions for 1 minute. The total run time was 4.7 min. Mass transitions of *m/z* 203 → 161 (KIC), 206 → 161 (KIC-d3), 189 → 174 (KIV), 194 → 178 (KIV-5C13), 203 → 174 (KMV), and 211 → 177 (KMV-d8) were monitored in a positive ion electrospray ionization mode.

Fecal DNA preparation and sequencing: Fecal DNA was collected and aliquoted as described (3). DNA was recovered from approximately 200mg of fecal material using the DNeasy Power Soil Pro kit (Qiagen). Bacterial community composition in DNA samples was characterized by amplification of the V4 variable region of the 16S rRNA gene by polymerase chain reaction using barcoded forward primer 515 and reverse primer 806 following the Earth Microbiome

Project protocol (http://www.earthmicrobiome.org/). Concentration of the PCR products was measured using a Qubit dsDNA HS assay kit (ThermoFisher, Q32854) and a Promega GloMax plate reader. Equimolar 16S rRNA gene PCR products for up to 384 samples were pooled prior to sequencing. Sequencing was performed by the Duke Sequencing and Genomic Technologies (SGT) Shared Resource on an Illumina NovaSeq instrument configured for 250 base-pair paired-end sequencing runs on a S Prime lane. Fecal DNA extraction, 16S rRNA library preparation, and sequencing, was performed at the Duke Microbiome Core laboratories. Taxa were identified using DADA2 (6) and resulting data was analyzed as described in the Statistical Analysis section.

Fecal DNA from a subset of 396 samples was submitted for metagenomic sequencing at the Novogene Corporation, Inc., using the following protocol. Genomic DNA was randomly fragmented by sonication, then DNA fragments were end polished, A-tailed, and ligated with the full-length adapters of Illumina sequencing, and followed by further PCR amplification with P5 and indexed P7 oligos. The PCR products as the final construction of the libraries were purified with AMPure XP system. Then libraries were checked for size distribution by Agilent 2100 Bioanalyzer (Agilent Technologies, CA, USA) and quantified by real-time PCR to meet the criteria of 3 nM. The qualified libraries were fed into Illumina NovaSeq sequencers after pooling according to effective concentration and expected data volume. Raw sequenced reads were then filtered to remove reads that contained adapters, reads containing greater than 10% unidentified bases, and reads with a quality score <5, or over 50% of the total bases. This resulted in an average of $3.6x10^7$ reads and 5.3Gb of data per sample.

Metagenome associated genome assembly and analysis: For construction of metagenome assembled genomes (MAG) and subsequent analysis, duplicates were removed and low quality bases and residual adapter sequences were trimmed from the metagenomic reads using prinseq-lite (v0.20.4, with argument -derep 1) (7) and Trimmomatic (v0.39, with argument LEADING:3 TRAILING:3 SLIDINGWINDOW:4:15 MINLEN:36) (8), respectively. Host reads were then identified by mapping to the CHM13 (v2.0) human reference genome (9) using Bowtie2 (v2.2.9, with argument --very-sensitive) (10) and removed. De-hosted reads were assembled de novo for each sample using megahit (v1.0.6) (11, 12) with default parameters. MAGs were then reconstructed for each sample using BASALT (v1.1.0, with arguments -m 90 -t 4 --min-cpn 50 --max-ctn 10 --refinepara quick) (13). Highly similar MAGs were identified using dRep (v3.6.2, with argument --S_algorithm fastANI -sa 0.99 -nc 0.5) (14). One MAG was selected to represent each dRep cluster, prioritizing the MAG with the highest genome completeness according to checkM (v1.1.2) (15). If multiple MAGs tied for the highest completeness, the MAG with the lowest contamination (according to checkM v1.1.2) was selected. 7109 representative MAGs selected based on the above criterion were taxonomically annotated using GTDB-TK (v1.6.0) (16). To estimate the relative abundance of the representative MAGs, metagenomic reads were mapped back to the representative MAGs using Bowtie2 (v2.2.9, with arguments --very-sensitive -k 10). Pathoscope (v2.0.6) (17) was used to probabilistically resolve ambiguous alignments and estimate the relative abundance ("Final Guess") of the MAGs.

References:

1. Armstrong SC, Windom M, Bihlmeyer NA, Li JS, Shah SH, Story M, et al. Rationale and design of "Hearts & Parks": study protocol for a pragmatic randomized clinical trial of an integrated clinic-community intervention to treat pediatric obesity. *BMC Pediatr.* 2020;20(1):308.

2. Matthews DR, Hosker JP, Rudenski AS, Naylor BA, Treacher DF, and Turner RC. Homeostasis model assessment: insulin resistance and beta-cell function from fasting plasma glucose and insulin concentrations in man. *Diabetologia.* 1985;28(7):412-9.
3. McCann JR, Bihlmeyer NA, Roche K, Catherine C, Jawahar J, Kwee LC, et al. The Pediatric Obesity Microbiome and Metabolism Study (POMMS): Methods, Baseline Data, and Early Insights. *Obesity (Silver Spring).* 2021;29(3):569-78.
4. White PJ, Lapworth AL, An J, Wang L, McGarrah RW, Stevens RD, et al. Branched-chain amino acid restriction in Zucker-fatty rats improves muscle insulin sensitivity by enhancing efficiency of fatty acid oxidation and acyl-glycine export. *Mol Metab.* 2016;5(7):538-51.
5. Olson KC, Chen G, and Lynch CJ. Quantification of branched-chain keto acids in tissue by ultra fast liquid chromatography-mass spectrometry. *Anal Biochem.* 2013;439(2):116-22.
6. Callahan BJ, McMurdie PJ, Rosen MJ, Han AW, Johnson AJ, and Holmes SP. DADA2: High-resolution sample inference from Illumina amplicon data. *Nat Methods.* 2016;13(7):581-3.
7. Schmieder R, and Edwards R. Quality control and preprocessing of metagenomic datasets. *Bioinformatics.* 2011;27(6):863-4.
8. Bolger AM, Lohse M, and Usadel B. Trimmomatic: a flexible trimmer for Illumina sequence data. *Bioinformatics.* 2014;30(15):2114-20.
9. Nurk S, Koren S, Rhie A, Rautiainen M, Bzikadze AV, Mikheenko A, et al. The complete sequence of a human genome. *Science.* 2022;376(6588):44-53.
10. Langmead B, and Salzberg SL. Fast gapped-read alignment with Bowtie 2. *Nat Methods.* 2012;9(4):357-9.
11. Li D, Luo R, Liu CM, Leung CM, Ting HF, Sadakane K, et al. MEGAHIT v1.0: A fast and scalable metagenome assembler driven by advanced methodologies and community practices. *Methods.* 2016;102:3-11.
12. Li D, Liu CM, Luo R, Sadakane K, and Lam TW. MEGAHIT: an ultra-fast single-node solution for large and complex metagenomics assembly via succinct de Bruijn graph. *Bioinformatics.* 2015;31(10):1674-6.
13. Qiu Z, Yuan L, Lian CA, Lin B, Chen J, Mu R, et al. BASALT refines binning from metagenomic data and increases resolution of genome-resolved metagenomic analysis. *Nat Commun.* 2024;15(1):2179.
14. Olm MR, Brown CT, Brooks B, and Banfield JF. dRep: a tool for fast and accurate genomic comparisons that enables improved genome recovery from metagenomes through de-replication. *ISME J.* 2017;11(12):2864-8.
15. Parks DH, Imelfort M, Skennerton CT, Hugenholtz P, and Tyson GW. CheckM: assessing the quality of microbial genomes recovered from isolates, single cells, and metagenomes. *Genome Res.* 2015;25(7):1043-55.
16. Chaumeil PA, Mussig AJ, Hugenholtz P, and Parks DH. GTDB-Tk: a toolkit to classify genomes with the Genome Taxonomy Database. *Bioinformatics.* 2019;36(6):1925-7.
17. Hong C, Manimaran S, Shen Y, Perez-Rogers JF, Byrd AL, Castro-Nallar E, et al. PathoScope 2.0: a complete computational framework for strain identification in environmental or clinical sequencing samples. *Microbiome.* 2014;2:33.

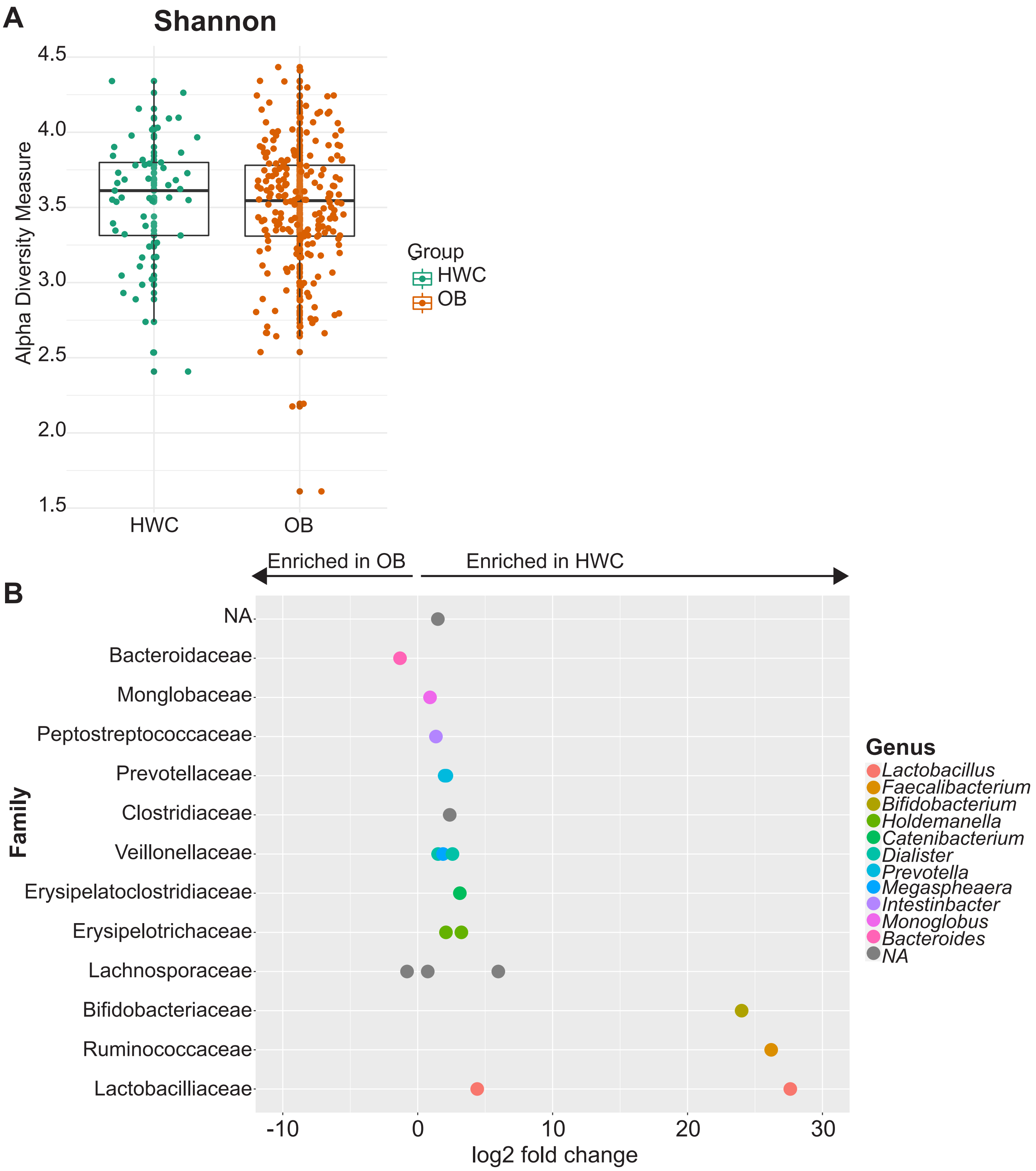

Supplemental Figure 1. 16S rRNA gene sequencing reveals similar fecal microbiome diversity between HWC and OB cohorts at baseline. **(A)** Alpha diversity metrics of gut microbial taxa as measured by 16S rRNA gene high throughput sequencing. HWC, healthy weight control; OB, adolescents with obesity. **(B)** Deseq2 measure of differential abundance at the family and genus level, when available, of microbial taxa comparing HWC and OBgroups.

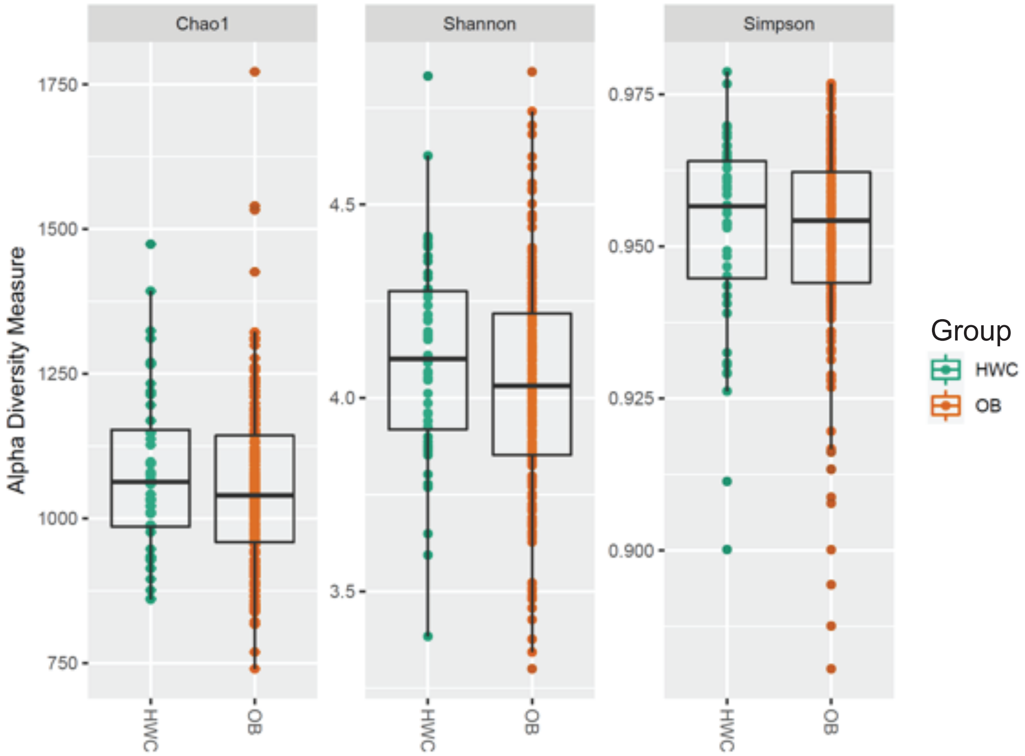

	p-value	Kruskal-Wallis chi-squared
Chao1	0.279628364997128	1.16889519261103
se.chao1	NaN	NaN
Shannon	0.118436952574392	2.43786996603581
Simpson	0.407967447520968	0.684716157205344

Supplemental Figure 2. Shotgun DNA sequencing on a subset of fecal samples resulted in similar alpha diversity measures between OB and HWC cohorts at baseline. A table of statistical analysis result values and p-values are also shown.

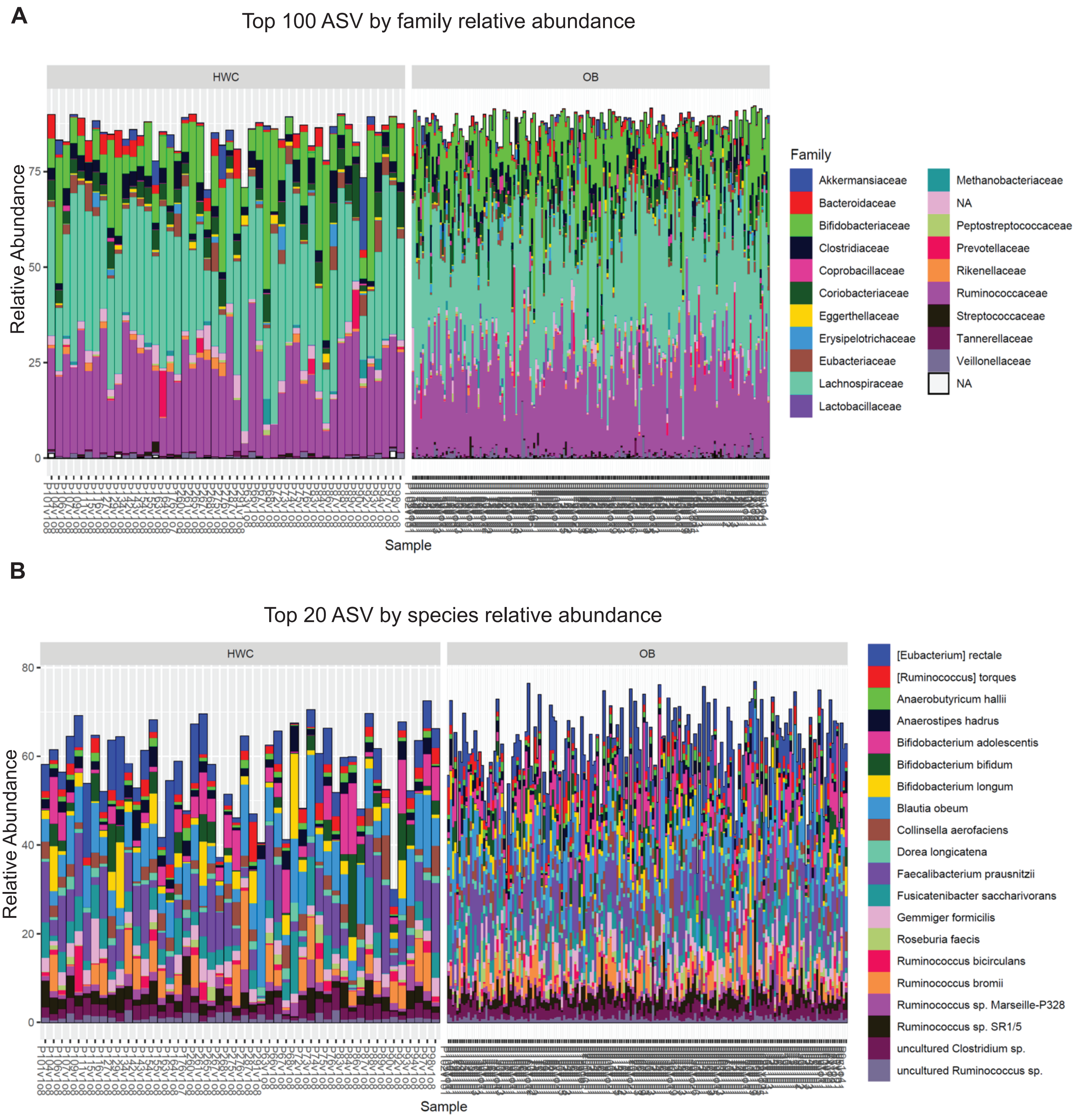

Supplemental Figure 3. Shotgun DNA sequencing demonstrated similar species and family community structure between OB and HWC cohorts at baseline. The top 100 most abundant families **(A)** and the top 20 most abundant species **(B)** from fecal metagenomic sequencing results are shown.

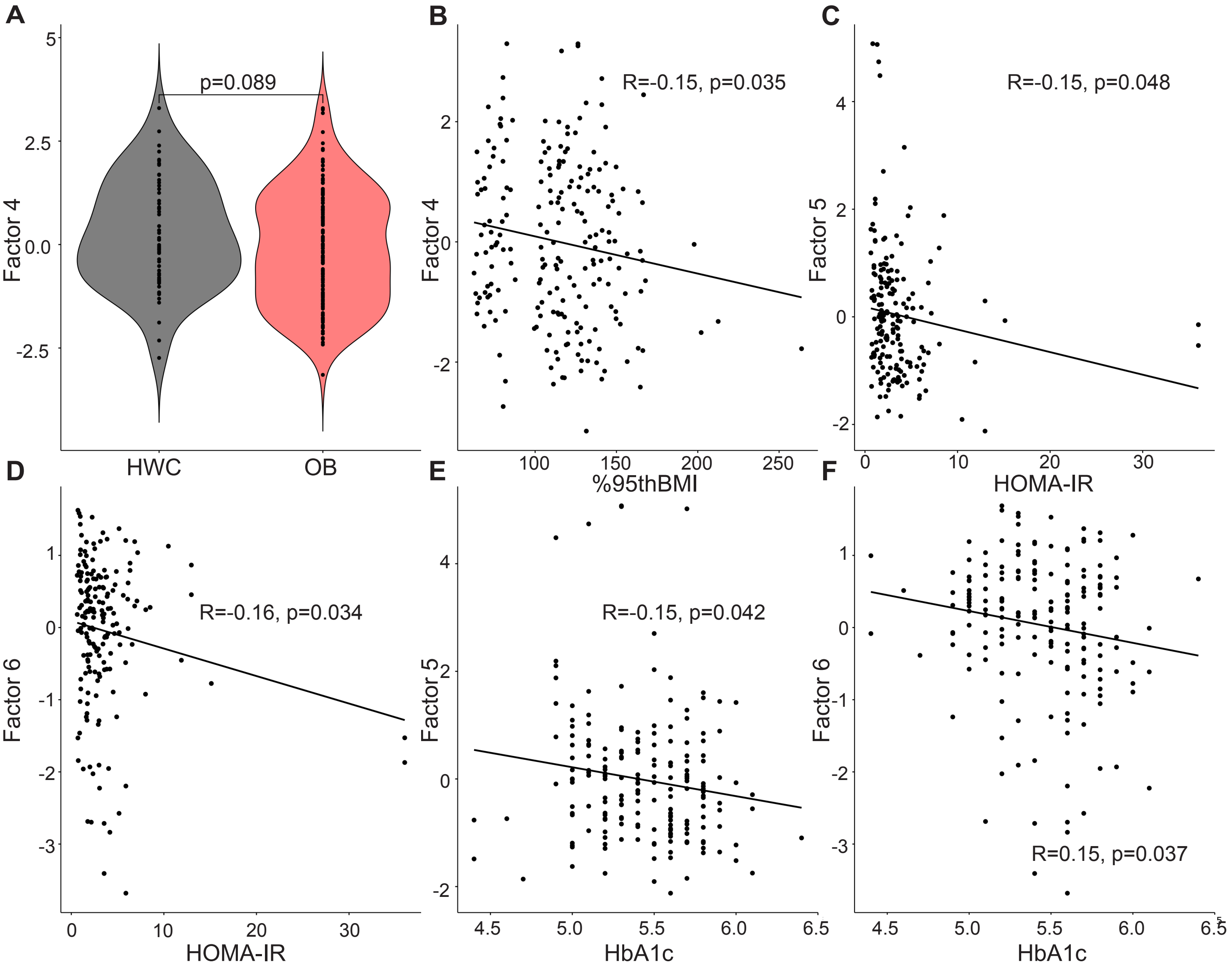

Supplemental Figure 4. Factor analysis suggests clusters of co-varying microbial taxa and functional gene content associate with clinical measures of health. Microbial taxa and gene content were clustered based on co-variance using hierarchical clustering. **(A)** Overall cluster member abundance from Factor 4 between the HWC and OB cohorts. Clusters with signficant assocations by linear regression with **(B)** %95thBMI, **(C-D)** HOMA-IR or **(E-F)** HbA1c are shown. Taxa and functional components that making up each of the microbial factors are identified in Supplemental Data.

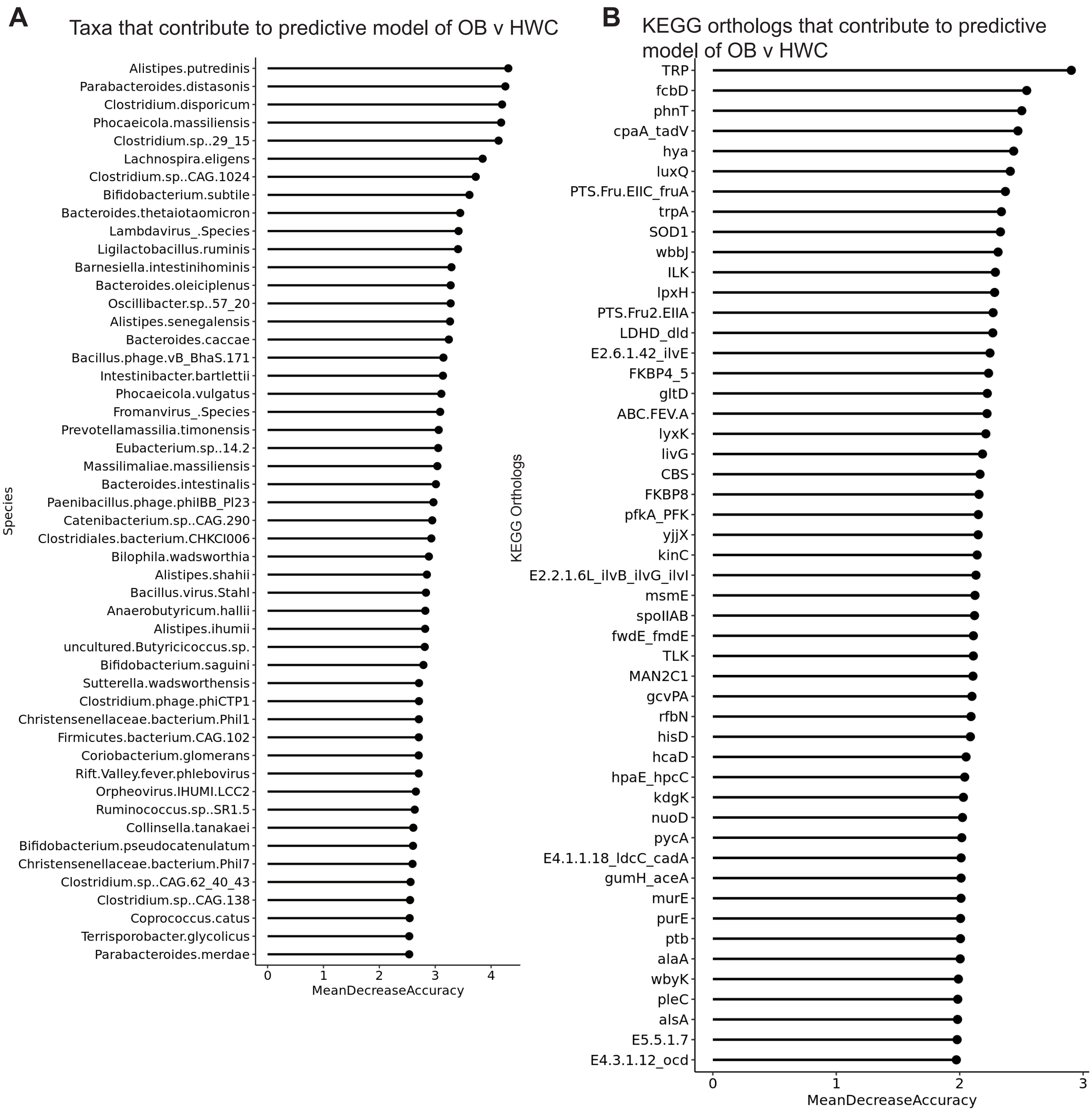

Supplemental Figure 5. Random Forests machine learning analysis suggests (A) microbial taxa or (B) KEGG functional orthologs that contribute to model prediction of OB v HWC status. "Mean decrease accuracy" score indicates the weight that each variable contributes to the accuracy of the predictive algorithm.

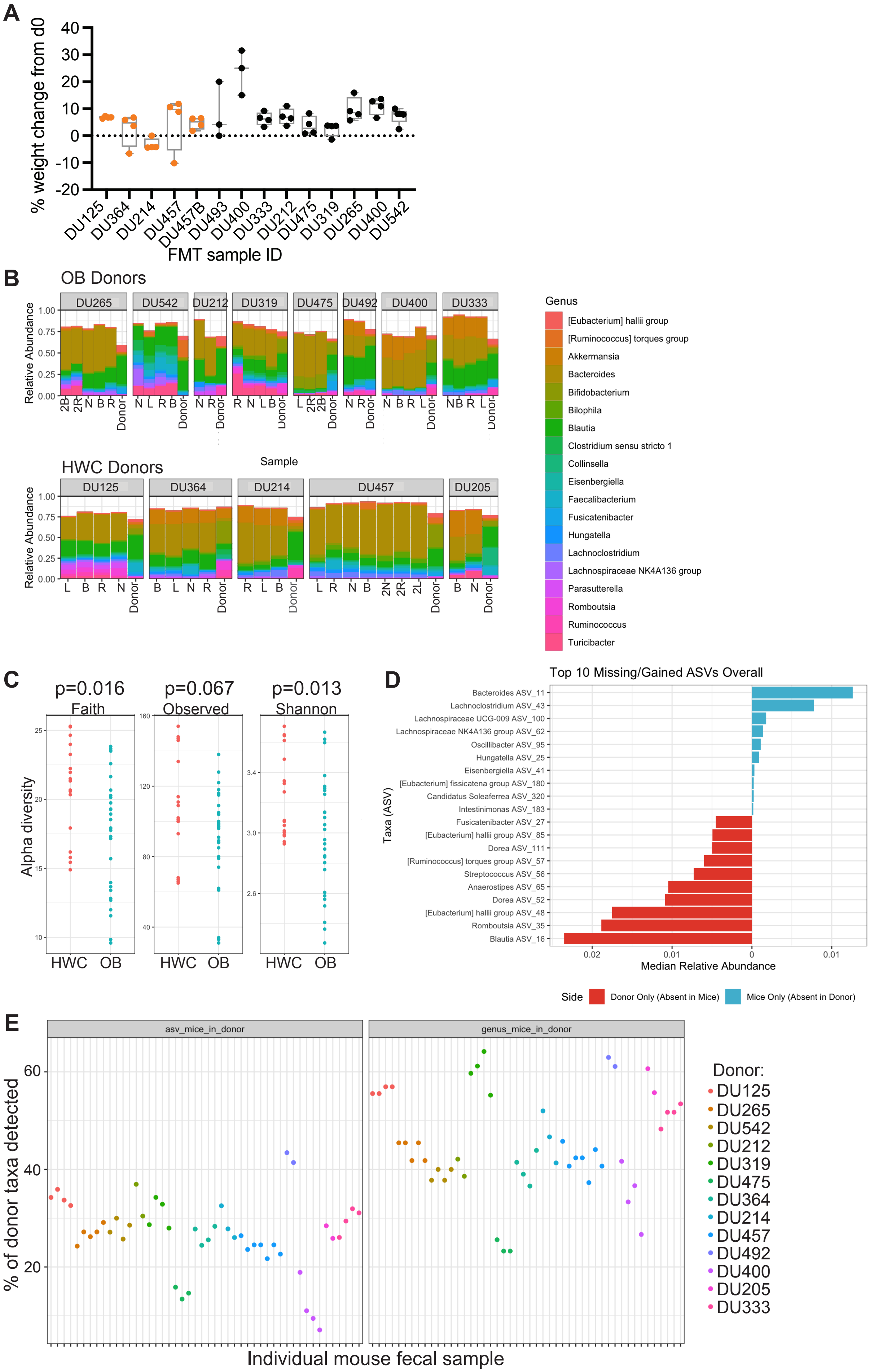
A
% weight change from d0
40
30
20
10
0
-10
-20
DU125
DU364
DU214
DU457
DU457B
DU493
DU400
DU333
DU212
DU475
DU319
DU265
DU400
DU542
FMT sample ID
B
OB Donors
DU265
DU542
DU212
DU319
DU475
DU492
DU400
DU333
Relative Abundance
1.00
0.75
0.50
0.25
0.00
Donor
Sample
HWC Donors
DU125
DU364
DU214
DU457
DU205
Genus
[Eubacterium] hallii group
[Ruminococcus] torques group
Akkermansia
Bacteroides
Bifidobacterium
Bilophila
Blautia
Clostridium sensu stricto 1
Collinsella
Eisenbergiella
Faecalibacterium
Fusicatenibacter
Hungatella
Lachnoclostridium
Lachnospiraceae NK4A136 group
Parasutterella
Romboutsia
Ruminococcus
Turicibacter
C
p=0.016
Faith
p=0.067
Observed
p=0.013
Shannon
Alpha diversity
HWC
OB
D
Top 10 Missing/Gained ASVs Overall
Bacteroides ASV_11
Lachnoclostridium ASV_43
Lachnospiraceae UCG-009 ASV_100
Lachnospiraceae NK4A136 group ASV_62
Oscillibacter ASV_95
Hungatella ASV_25
Eisenbergiella ASV_41
[Eubacterium] fissicatena group ASV_180
Candidatus Soleaferrea ASV_320
Intestinimonas ASV_183
Fusicatenibacter ASV_27
[Eubacterium] hallii group ASV_85
Dorea ASV_111
[Ruminococcus] torques group ASV_57
Streptococcus ASV_56
Anaerostipes ASV_65
Dorea ASV_52
[Eubacterium] hallii group ASV_48
Romboutsia ASV_35
Blautia ASV_16
Taxa (ASV)
Median Relative Abundance
Side
Donor Only (Absent in Mice)
Mice Only (Absent in Donor)
E
asv_mice_in_donor
genus_mice_in_donor
% of donor taxa detected
Individual mouse fecal sample
Donor:
DU125
DU265
DU542
DU212
DU319
DU475
DU364
DU214
DU457
DU492
DU400
DU205
DU333

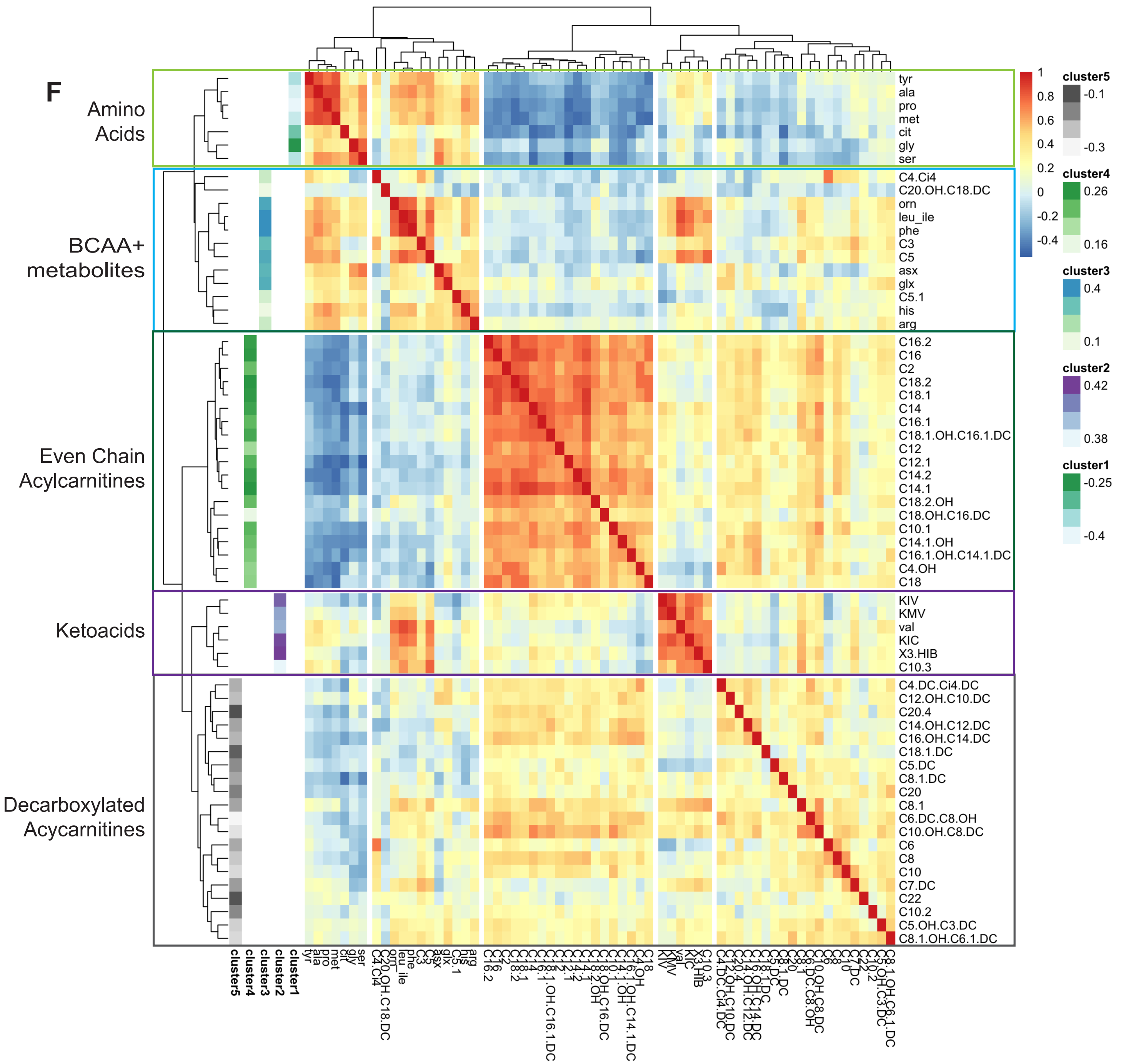

Supplemental Figure 6. Donor status of fecal microbiome did not affect weight gain of recipient mice but did affect resulting fecal microbiome diversity. (A) Percent weight change for germ-free mice 2 weeks after receiving a fecal microbiome transplant from adolescents in the POMMS cohort. Each dot represents a single animal. Box plots represent mean and 1 standard deviation. Orange, HWC donor. Black, donors with OB. **(B)** Input gavage donor slurry (Donor) and mouse fecal microbiome community composition at 2 weeks post gavage as measured by 16S rRNA gene high throughput sequencing. R, N, L, B, 2R, 2R, 2N, 2B are individual animal tag labels. **(C)** Mouse fecal microbiome alpha diversity measures at 2 weeks post FMT gavage. **(D)** Percent of donor taxa by ASV or genus detected in fecal 16SrRNA sequencing at 2 weeks post colonization. Each dot represents an individual mouse colored by donor source. **(E)** Top 10 ASV recovered in fecal mouse 16S rRNA sequencing but not in donor gavage, and to 10 ASV in donor gavage that were not detected in fecal samples. **(F)** Results of hierarchical clustering of mouse serum metabolites for comparison with gut microbial taxa as referenced in main Figure 5G. Heatmap scale (red to blue) demonstrates intensity and direction of assocation, and Cluster color scales describe strength of association within per metabolite within each metabolite cluster.

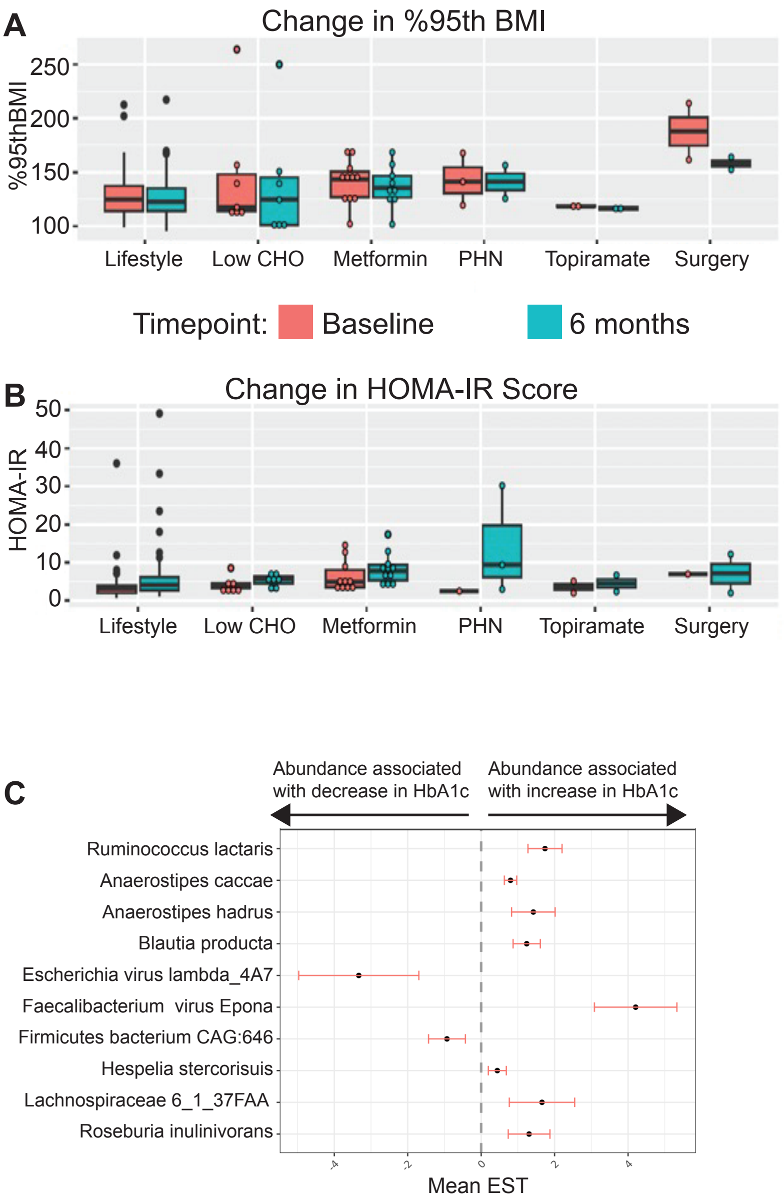
A
Change in %95th BMI
%95thBMI
250
200
150
100
Lifestyle
Low CHO
Metformin
PHN
Topiramate
Surgery
Timepoint:
Baseline
6 months
B
Change in HOMA-IR Score
HOMA-IR
50
40
30
20
10
0
Lifestyle
Low CHO
Metformin
PHN
Topiramate
Surgery
C
Abundance associated with decrease in HbA1c
Abundance associated with increase in HbA1c
Ruminococcus lactaris
Anaerostipes caccae
Anaerostipes hadrus
Blautia producta
Escherichia virus lambda_4A7
Faecalibacterium virus Epona
Firmicutes bacterium CAG:646
Hespelia stercorisuis
Lachnospiraceae 6_1_37FAA
Roseburia inulinivorans
-4
-2
0
2
4
Mean EST

D

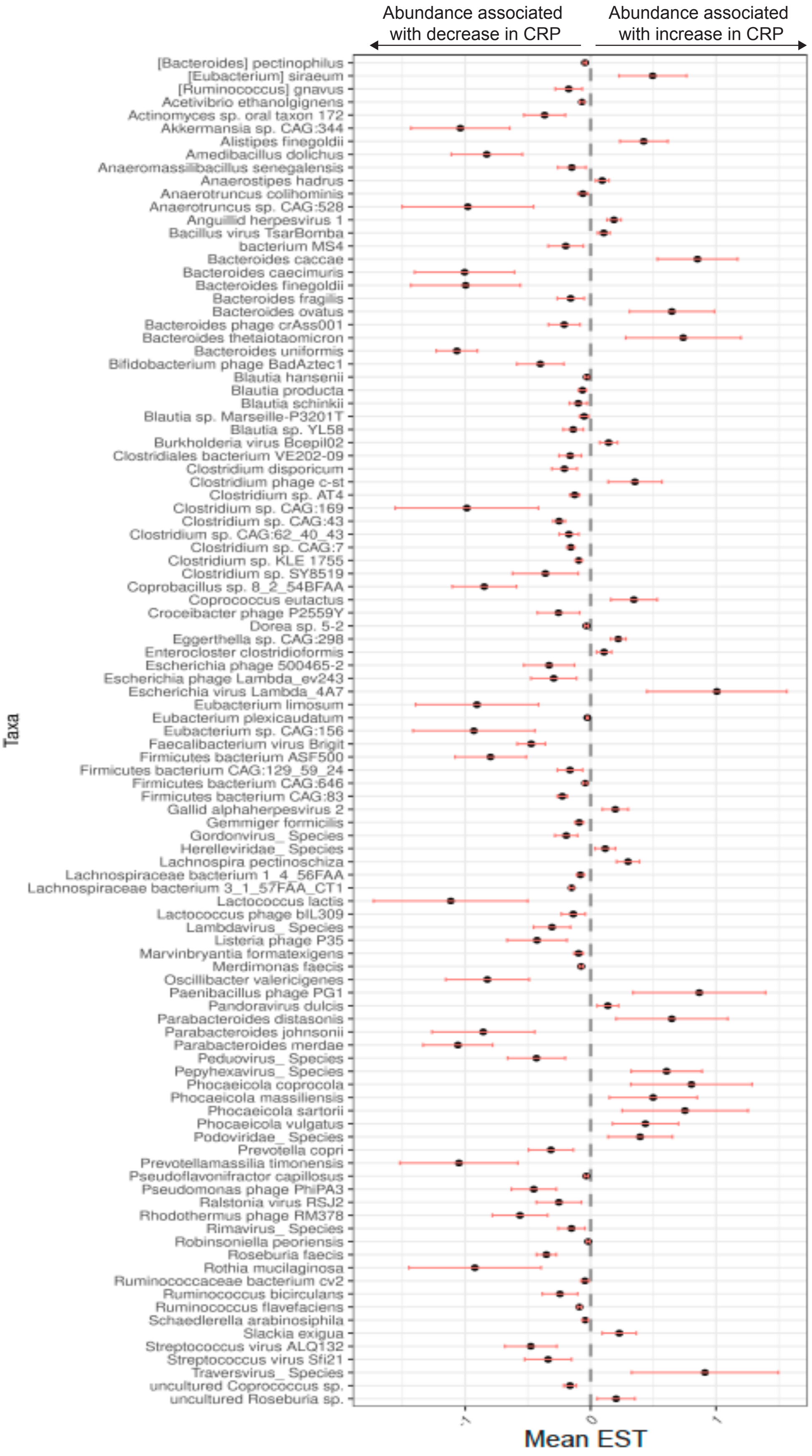

Supplemental Figure 7. Most adolescents saw an increase in HOMA-IR over the observational period, and several baseline gut microbial taxa were significantly associated with changes in HbA1C and CRP in the OB cohort. Mean and standard deviation of **(A)** %95th BMI and **(B)** HOMA-IR score at baseline (salmon) and 6 months (teal) for each treatment group within the observational trial. Baseline fecal microbiome taxa that were significantly associated with changes in **(C)** HbA1c or **(D)** C-reactive protein (CRP) levels over the observational period in the OB cohort. Abbreviations: CHO, carbohydrate; PHN, Phentermine.

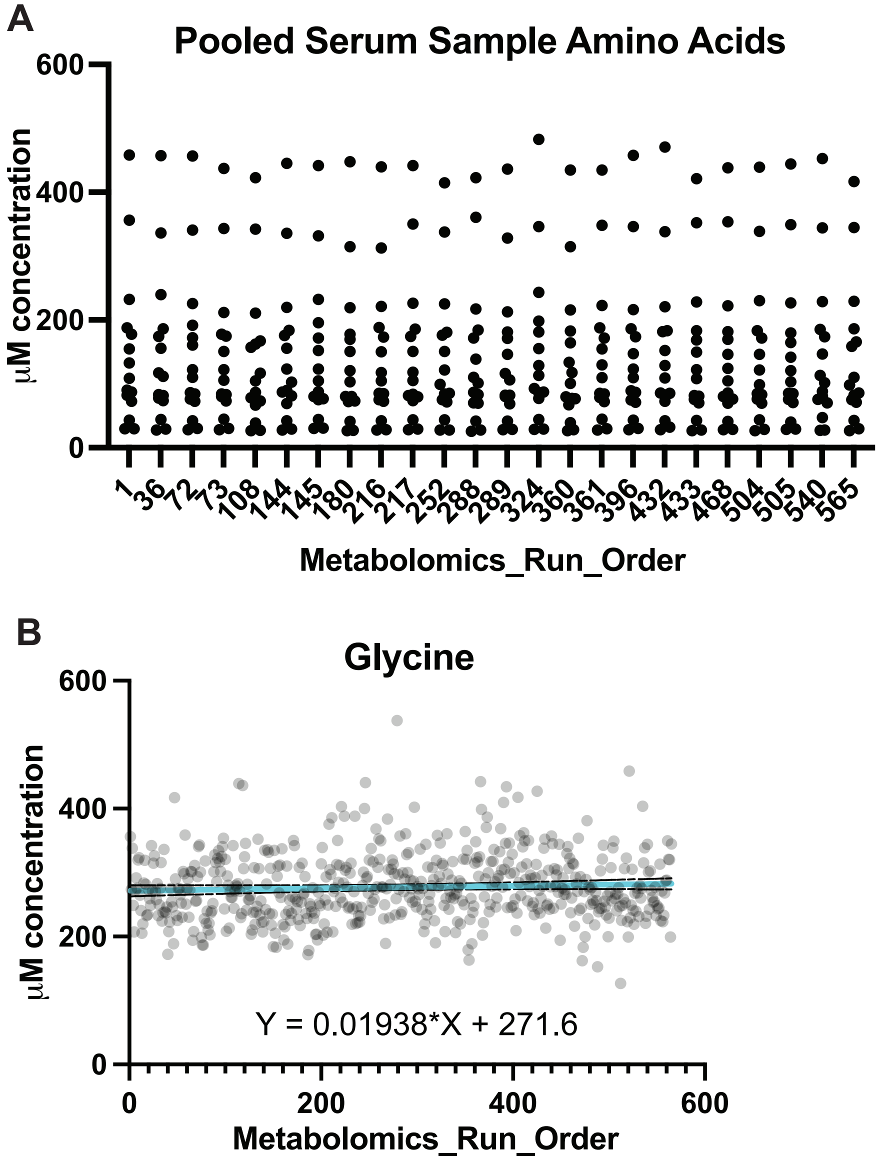

Supplemental Figure 8. Run order plots demonstrate minimal drift in sample measurement data. Raw concentrations of 17 amino acid measurements from a pooled serum sample measured with every sample batch **(A)** and all glycine measurements **(B)** were plotted to assess variation across run order. The equation of the line and shaded confidence interval is shown and was not significantly different from 0 (p=0.2).
